# Skin autofluorescence predicts cardio-renal outcome in type 1 diabetes: a longitudinal study

**DOI:** 10.1186/s12933-016-0448-8

**Published:** 2016-09-01

**Authors:** Fritz-Line Vélayoudom-Céphise, Kalina Rajaobelina, Catherine Helmer, Sovanndany Nov, Emilie Pupier, Laurence Blanco, Marie Hugo, Blandine Farges, Cyril Astrugue, Henri Gin, Vincent Rigalleau

**Affiliations:** 1 Nutrition Diabetology Unit, CHU of Bordeaux, Haut Lévêque Hospital, Pessac, France; 2 Research Group Clinical Epidemiology and Medicine ECM/L.A.M.I.A, EA 4540, University of Antilles, Guadeloupe, France; 3 Department of Diabetology-Endocrinology, University Hospital of Pointe-à-Pitre, Guadeloupe, France; 4 INSERM, ISPED, Centre INSERM U897-Epidemiology-Biostatistics, Bordeaux, France

**Keywords:** Type 1 diabetes, Skin autofluorescence, Macrovascular events, Albumin excretion rate, Estimated glomerular filtration rate

## Abstract

**Background:**

We aimed to analyze the relationships between skin autofluorescence (SAF) and incident macrovascular events and renal impairment after 4 years of follow-up in patients with type 1 diabetes (T1D).

**Methods:**

Two hundred and forty-three patients (51.2 ± 16.7 years old) with T1D participated. SAF was measured by AGE-Reader-TM at inclusion. Macrovascular events (MVE), estimated glomerular filtration rate (eGFR) and urinary albumin excretion rate (AER) were recorded then and 4 years later. Multivariate logistic regression was used to analyze the relationships between SAF and incident MVE and renal profile 4 years later.

**Results:**

Patients with incident MVE had a higher SAF (p = 0.003). SAF predicted incident MVE after adjustment for age, sex, body mass index, tobacco, diabetes duration, hypertension, HbA_1c_, AER, eGFR (OR 4.84 [95 % CI 1.31–17.89], p = 0.018). However, this relation was no longer significant after adjustment for history of MVE. An inverse relation was found between SAF and incident eGFR (p = 0.0001). Patients with incident eGFR <60 ml/min/1.73 m^2^ had a SAF higher than patients with normal eGFR. After adjustment for the previous criteria, SAF remained associated with the risk of impaired incident eGFR (OR 7.42 [95 % CI 1.59–34.65], p = 0.018). No relation was found between SAF and increased AER 4 years later.

**Conclusions:**

SAF predicts MVE in patients with T1D, adjusted for cardiovascular risk factors but the most powerful predictive factor remains history of MVE. SAF also predicts eGFR impairment, adjusted for initial AER and renal function. SAF could be a useful non-invasive tool for estimating risk of cardiovascular or renal impairment in patients with T1D.

## Background

Mainly due to cardiovascular disease, patients with type 1 diabetes (T1D) have an increased mortality rate as compared to the general population [1], especially when their diabetes is complicated by diabetic chronic kidney disease (CKD), with abnormal albumin excretion rates (AER) or reduced glomerular filtration rates (GFR) [2].

Advanced glycation end-products (AGEs) are produced by the non-enzymatic Maillard reaction between reducing sugars and proteins, lipids or nucleic acids, forming Schiff bases and Amadori products. Further oxidation and dehydration give rise to numerous AGEs. They can be exogenously ingested from food, or endogenously produced in situations of hyperglycaemia and oxidative stress [3]. In this context, AGEs are suspected to play an important role in this cardio-renal risk, as they promote inflammation and endothelial dysfunction [4]. Their accumulation is increased in CKD, due to reduced clearance of their precursors [5]. High levels of circulating AGEs predict later mortality and cardiovascular events in T1D [6], and high levels of AGEs in skin biopsies predict microangiopathy [7] and sub-clinical cardiovascular disease in the EDIC study [8]. But the complex time consuming analysis of AGEs, and the invasiveness of skin biopsies preclude the use of AGEs for prognosis in a normal clinical routine.

This may change with the development of non-invasive methods for measuring AGEs, based on their fluorescent properties: the skin autofluorescence (SAF) is well correlated with the dermal concentrations of AGEs [9], its measurement is safe, quick and simple. SAF has been shown to predict later microangiopathy [10] and cardiovascular events [11] in patients with type 2 diabetes mellitus (T2D). To date no longitudinal study has been performed in T1D.

We measured SAF in 300 patients with T1D during year 2009. Our baseline results, which show that SAF was related to retinopathy, nephropathy and long term past glucose control, have been published elsewhere [12]. In the present study, we investigated whether SAF was related to their cardio-renal outcome within 4 years of follow-up: cardiovascular events, estimated GFR (eGFR), and AER. Data were available in 243 patients after 4 years of follow-up.

## Patients and methods

### Design and patients

We analyzed the data from two hundred and forty-three patients with T1D included during the year 2009 after written informed consent. The following data were recorded at inclusion, on the day of SAF measurement: age, sex, body mass index (BMI), duration of diabetes, hypertension (defined by a blood pressure ≥140/90 mm Hg and/or treatment with antihypertensive drug) and smoking history. Smokers included patients with past and current smoking history. The biological data recorded included HbA_1c_ levels, serum creatinine levels, eGFR calculated with the CKD-EPI formula and AER.

### Skin autofluorescence (SAF)

The accumulation of advanced glycation end-products was evaluated from SAF measured by illumination of 1 cm^2^ of the forearm skin by the AGE reader SU 2.3 (DiagnOptics BV, Groningen, the Netherlands) at inclusion. This technique uses fluorescent properties of some AGEs to estimate AGE accumulation in the skin [9]. The patients with Fitzpatrick phototypes V and VI were not included due to their skin pigmentation, which had ultraviolet reflectance of <10 %. SAF values were calculated by dividing the mean emitted light intensity (excitation light source ranging from 300 to 420 nm) by the mean reflected excitation light intensity from the skin (over 300 to 420 nm). Results were the mean of 3 consecutive measurements of SAF and were expressed in arbitrary units (AU).

### Outcomes

The patients were prospectively followed-up and incident macrovascular events (MVE) and renal outcome were recorded after 4 years. MVE were defined as myocardial infarction, stroke, gangrene, or a revascularization procedure for coronary, carotid or lower limb arteries.

The renal outcome analysis included AER (mg/24 h) and renal function (eGFR, ml/min per 1.73 m^2^), measured 4 years after inclusion.

### Statistical methods

The data are presented as mean ± SD for continuous variables and as numbers (percentages) for categorical variables. Student’s *t* test was used to compare continuous variables.

A logistic regression was used to investigate the associations between SAF and MVE, after adjustment for the following co-variables: age, sex, BMI, hypertension, duration of diabetes and tobacco. A multivariate regression analysis was used to investigate the associations between SAF and later AER and eGFR, after adjustment for the following co-variables: age, sex, hypertension, duration of diabetes and tobacco. We also performed complementary stepwise backwards multivariate logistic regression analyses. All analyses were performed with SAS Statistical Package release 9.3 (SAS Institute, Cary, NC) and results were considered significant when p < 0.05.

## Results

### Characteristics of the population

Mean age of the 243 patients at inclusion was 51.2 ± 16.7 years and 58.9 % of patients were men. Duration of diabetes was 21.4 ± 13.8 years. Means of eGFR and AER at inclusion were 86.25 ± 26.58 ml/mn/1.73 m^2^ and 54 ± 220 mg/24 h respectively.

Twenty-four (9.9 %) of patients had a history of MVE and 53 (22.8 %) had CKD at baseline including 15.6 % (N = 37) of patients with AER ≥30 mg/24 h and 14.4 % (N = 34) with eGFR <60 ml/mn/1.73 m^2^.

Mean SAF value was 2.13 ± 0.58 AU at baseline related to age, gender, duration of diabetes and eGFR (not to BMI).

### Skin autofluorescence and cardiovascular outcome

SAF levels were higher in patients with history of MVE at baseline (2.58 ± 0.71 AU) compared to patients free of MVE (2.09 ± 0.54 AU, p = 0.003). This difference persisted after adjustment for sex, age, BMI, tobacco, diabetes duration, arterial hypertension and HbA_1c_ (B = 0.26 [0.03–0.50], p = 0.027), but not after adjustment for AER and eGFR (B = 0.17 [−0.06 to 0.40], p = 0.149).

The 14 patients who experienced incident MVE during the 4 year follow-up had a higher SAF at baseline (2.59 ± 0.72) compared to the others (2.11 ± 0.56, p = 0.002). The multivariate regression analysis revealed that SAF was significantly associated with incident MVE (OR 4.84 [95 % CI 1.31–17.89], p = 0.02), after adjustment for age, sex, BMI, tobacco, arterial hypertension, duration of diabetes, HbA1C, eGFR <60 ml/min/1.73 m^2^ and microalbuminuria (AER ≥30 mg/24 h). This relationship was not significant after taking into account history of MVE, the strongest predictor of incident MVE (Table 1). By complementary stepwise backward multivariate logistic regression analysis, the variables related to incident MVE were the baseline history of MVE and the BMI. Tobacco and eGFR <60 ml/min/1.73 m^2^ tended to be related (p = 0.055 and 0.068 respectively).

**Table 1:** Relationships between skin autofluorescence and macrovascular events after 4 years of follow-up in subjects with type 1 diabetes

	Univariate analysis OR (CI 95 %)	p	Multivariate analysis OR (CI 95 %) with adjustment for history of MVE: N = 214	p	Multivariate analysis OR (CI 95 %) without adjustment for history of MVE: N = 214	p
SAF	3.22 (1.45–7.13)	0.004	2.98 (0.77–11.47)	0.11	4.84 (1.31–17.89)	0.02
Age	1.06 (1.02–1.10)	0.006	1.03 (0.97–1.09)	0.40	1.04 (0.98–1.10)	0.19
Sex	0.37 (0.10–1.37)	0.14	0.69 (0.11–4.26)	0.69	0.34 (0.07–1.78)	0.20
BMI^a^	1.13 (1.01–1.27)	0.036	1.18 (0.98–1.40)	0.08	1.13 (0.98–1.31)	0.10
Arterial hypertension	8.17 (1.79–37.32)	0.007	2.27 (0.35–14.74)	0.39	3.50 (0.65–18.94)	0.15
Duration of diabetes	1.05 (1.01–1.10)	0.007	1.00 (0.95–1.07)	0.88	1.02 (0.97–1.07)	0.49
HbA_1c_^a^	0.82 (0.46–1.47)	0.51	0.65 (0.23–1.81)	0.41	0.84 (0.40–1.77)	0.65
Tobacco^a^	0.47 (0.10–2.14)	0.33	0.12 (0.01–1.32)	0.08	0.43 (0.07–2.60)	0.36
Albumin excretion rate ≥30 mg/24 h^a^	3.32 (1.04–10.53)	0.04	2.452 (0.40–15.07)	0.33	2.31 (0.47–11.32)	0.30
eGFR <60 mL/min/1.73 m^2^ at baseline^a^	1.68 (0.44–6.37)	0.45	0.14 (0.02–1.26)	0.08	0.27 (0.05–1.64)	0.16
MVE at baseline	25.68 (7.64–86.30)	<0.0001	21.93 (3.85–124.81)	0.0005	–	–

*SAF* skin autofluorescence; *CKD* chronic kidney disease; *MVE* macrovascular events

^a^Missing data: BMI (n = 10); HbA_1c_ (n = 1); Tobacco (n = 13); Albumin Excretion Rate (n = 6); eGFR (n = 7)

### Skin autofluorescence and renal outcome

Mean SAF was higher in patients with impaired renal function (eGFR <60 ml/min/1.73 m^2^) at baseline compared to patients with normal eGFR (2.69 ± 0.67 vs 2.05 ± 0.51 AU, p < 0.0001). There was no difference between SAF of patients according to AER at baseline.

An inverse relation was found between SAF and eGFR 4 years later (r = −0.39, p = 0.0001).

SAF was higher in the 7 patients with incident impairment of eGFR (<60 ml/min/1.73 m^2^) compared to the others (2.53 ± 0.56 vs. 2.07 ± 0.50 AU, p < 0.0001). The result remained significant after adjustment for age, sex, BMI, tobacco, diabetes duration, hypertension, history of macrovascular disease, HbA_1c_, AER ≥30 mg/24 h and eGFR <60 ml/min/1.73 m^2^ at inclusion (OR = 7.42 [95 % CI 1.59–34.65], p = 0.01). Table 2 shows the results of logistic regression for the association between SAF and impaired eGFR, 4 years later. By complementary stepwise backwards multivariate logistic regression analysis, we found that three variables were related to a final eGFR <60 ml/min/1.73 m^2^: arterial hypertension, initial eGFR <60 ml/min/1.73 m^2^ and SAF. BMI also tended to be associated (p = 0.053).

**Table 2:** Relationships between skin autofluorescence and impaired eGFR (<60 mL/min/1.73 m^2^) after 4 years of follow-up in subjects with type 1 diabetes

	Univariate analysis OR (CI 95 %)	p	Multivariate analysis OR (CI 95 %) with adjustment for history of impaired eGFR <60 ml: N = 186	p
SAF	4.60 (2.11–10.00)	<0.0001	7.42 (1.59–34.65)	0.01
Age	1.05 (1.02–1.09)	0.002	1.03 (0.96–1.09)	0.43
Sex	0.94 (0.40–2.20)	0.89	1.06 (0.21–5.33)	0.95
BMI^a^	1.13 (1.02–1.25)	0.016	1.18 (0.99–1.41)	0.06
Arterial hypertension	4.56 (1.74–11.95)	0.002	6.29 (0.86–46.11)	0.07
Duration of diabetes	1.04 (1.01–1.07)	0.013	1.01 (0.95–1.07)	0.78
HbA1c^a^	0.86 (0.53–1.39)	0.55	0.80 (0.41–1.58)	0.52
Tobacco^a^	0.61 (0.20–1.88)	0.39	0.91 (0.15–5.40)	0.92
Albumin excretion rate ≥30 mg/24 h^a^	7.30 (2.87–18.57)	<0.0001	3.14 (0.56–17.61)	0.19
eGFR <60 ml/min/1.73 m^2^ at baseline^a^	55.93 (18.17–172.18)	0.035	48.04 (8.84–261.07)	<0.0001
History of MVE at baseline	3.37 (1.09–10.42)	<0.0001	0.40 (0.06–2.90)	0.37

^a^Missing data: BMI (n = 10); HbA_1c_ (n = 1); tobacco (n = 13); albumin excretion rate (n = 6); eGFR (n = 7); EpiCKD (n = 30)

We did not find any relation between SAF and increased AER 4 years later after complete adjustment including the history of increased AER (Table 3). By complementary stepwise backwards-multivariate logistic regression analysis, we found that three variables were related to a final AER ≥30 mg/24 h: age, initial eGFR <60 ml/min/1.73 m^2^ and initial AER ≥ 30 mg/24H.

**Table 3:** Relationships between skin autofluorescence and increased AER after 4 years of follow-up in subjects with type 1 diabetes

	Univariate analysis OR (CI 95 %)	p	Multivariate analysis OR (CI 95 %) with adjustment for history of AER: N = 168	p
SAF	1.44 (0.70–2.97)	0.32	0.35 (0.10–1.21)	0.10
Age	1.04 (1.01–1.07)	0.005	1.043 (1.00–1.09)	0.07
Sex	0.99 (0.45–2.19)	0.98	1.73 (0.51–5.89)	0.38
BMI^a^	1.08 (0.98–1.19)	0.11	0.98 (0.85–1.13)	0.78
Arterial hypertension	4.63 (1.88–11.42)	0.0009	1.61 (0.45–5.72)	0.46
Duration of diabetes	1.03 (1.00–1.06)	0.057	1.02 (0.97–1.06)	0.48
HbA1c^a^	1.05 (0.70–1.58)	0.80	1.08 (0.63–1.85)	0.79
Tobacco^a^	1.07 (0.42–2.71)	0.90	3.60 (0.93–13.94)	0.06
Albumin excretion rate ≥30 mg/24 h^a^	19.09 (7.27–50.13)	<0.0001	16.68 (4.38–63.66)	<0.0001
eGFR <60 ml/min/1.73 m^2^ at baseline^a^	9.67 (3.66–25.54)	<0.0001	7.02 (1.55–31.77)	0.01
MVE at baseline	4.72 (1.50–14.80)	0.0078	3.12 (0.56–17.37)	0.19

*SAF* skin autofluorescence; *CKD* chronic kidney disease; *MVE* macrovascular events

^a^Missing data: BMI (n = 10); HbA_1c_ (n = 1); tobacco (n = 13); albumin excretion rate (n = 6); eGFR (n = 7)

## Discussion

In our study of 243 patients with T1D, SAF measured during year 2009 relates to the risk of MVE and impaired eGFR 4 years later. Our study is the first to show a relation between SAF and incident MVE in T1D: as shown in Table 1, SAF predicts incident MVE during the four following years. This relation is not significant after adjustment for history of previous MVE, which is the most powerful predictor of incident MVE, but the fact that only SAF significantly relates to incident MVE when accounted for age, duration and control of diabetes, arterial hypertension, tobacco use and renal parameters is noteworthy. Moreover, this relationship remains significant after adjustment for AER and eGFR. Consequently, this result seems important, because diabetic nephropathy is a major predictor of cardiovascular risk in T1D [1, 2].

Furthermore, we found that SAF predicts later impairment of eGFR in our patients. This relation persists after adjustment to the history of impaired eGFR. By contrast to the relationship between SAF and MVE, incident impaired eGFR is predicted by SAF irrespective of renal function at baseline. We also showed that SAF predicts later eGFR <60 mL/min/1.73 m^2^ adjusted for initial AER. Otherwise, we did not find any relationships between SAF and AER after 4 years in contrast to eGFR. This result is not surprising as the studied population was followed in a university hospital with systematic preventive treatment of diabetic nephropathy including renin-angiotensin-system blockers. Moreover, the renal outcome of patients with T1D has been reported as being independent from AER changes that was more related to glycemic, blood pressure and lipid profile control [13]. The early loss of renal function in patients with T1D may begin before onset of proteinuria, although the mechanism involved in the changes of eGFR remains poorly understood. To our knowledge, no studies have noted whether renal impairment begins during microalbuminuria or during the normoalbuminuria state.

A prospective study about the role of SAF to predict cardiovascular events in patients with T2D has been reported [11], but such results have yet to be described in patients with T1D. Only cross sectional studies were performed in T1D and showed that SAF was associated with subclinical markers of macroangiopathy as intima-media thickness [14] and arterial stiffness [15, 16]. Furthermore, skin fluorescence could be measured with the SCOUT DM^®^ (VeraLight, Inc., Albuquerque, NM) another method for assessing intra-patient skin variation irrespective of the Fitzpatrick phototype. Skin intrinsic fluorescence could be related to the severity of coronary calcifications [17] and the intima-media thickness [18]. In 172 patients with long duration T1D, it was associated with a clinical history of coronary heart disease, but the relation lost significance after adjustment for nephropathy [19], similar to our finding at baseline. The EURODIAB study has already reported that increased levels of soluble receptor for advanced glycation end products (RAGE) were related to CV disease in T1D [20]. In addition, high circulating levels of AGEs were reported to predict incident CVD in T1D [6], and their concentrations in skin biopsies were related to coronary calcifications, left ventricular masses and progression of the intima-media thickness in the DCCT-EDIC study [8]. Consequently, SAF, a non-invasive tool for estimating accumulation of AGE could be linked to vascular modification and thus assess risk of MVE. However, the value of SAF as a surrogate marker to estimate tissues AGEs accumulation and to predict MVE is a matter of debate [21]. Indeed, SAF has been found to be correlated with skin levels of AGEs such as pentosidine, *N*-(carboxymethyl)lysine and *N*-carboxyethyl-lysine, and positively associated with age, diabetes duration, and mean glycated hemoglobin of the previous year in both type 1 and type 2 diabetic patients [22]. Moreover, a relation between SAF and cardiac tissue glycation has been reported [23]. SAF levels could depend on their tissue turnover, so it must be distinguished from the plasma concentrations of AGEs [21]. However, SAF remains a reliable clinical tool for evaluating tissue accumulation levels of AGEs. Indeed, tissue levels of AGEs estimated by skin fluorescence are a potential biomarker to evaluate vascular risks in patients with diabetes [17–19]. Finally, further longitudinal studies are needed to clarify whether SAF could be a predictor of the development and progression of long-term MVE. In the same way, the significance of the association between SAF and MVE after adjustment for AER and eGFR also seems important, since diabetic nephropathy is a major predictor of the cardiovascular risk in T1D [1, 2].

In this context, SAF has been shown to predict later microalbuminuria in patients with T2D [10], and the doubling of serum creatinine or the need for dialysis in CKD [24]. In the same way, cross-sectional studies performed in T1D have shown associations of SAF with AER and eGFR [12, 25]. Moreover, a longitudinal study was especially needed for renal markers, because high SAF may favor, but also may result from impaired GFR [26]. Our current results show that SAF predicts later eGFR <60 mL/min/1.73 m^2^, adjusted for initial AER and eGFR. This is consistent with the recent report that AGEs in skin biopsies related to later increase of serum creatinine in patients with both types of diabetes [27]. Because the relation between SAF and later low eGFR persists after adjustment to AER, we suggest that AGEs may play a role in the yet unexplained rapid decline in GFR observed in some patients with T1D, with [28] or without microalbuminuria [13]. Indeed, high serum levels of AGEs have been related to the progression of glomerular basal membrane in normoalbuminuric T1D patients [29]. Moreover, mean values of skin collagen linked AGEs such as furosine, carboxymethyl-lysine, pentosidine, and fluorescence were significantly higher in the patients with progression of nephropathy [7]. AGEs have been shown to be significant predictors of nephropathy, independently of the mean HbA1c [7].

The prediction of cardiorenal events from SAF may reflect its value as an integrator of long-term glucose control, as a marker of “metabolic memory”. In this context, we have recently shown that SAF better relates to plasma glucose levels, HbA_1c_ and renal function 10 years earlier than to their assessment at the time of SAF measurement in elderly patients from the general population [30]. The risk of microvascular complications like retinopathy has indeed been related more to ancient than recent HbA_1c_ in T1D [31]. In a previous study, we noted that SAF was better correlated with the previous years than present HbA_1c_ [12], in line with other authors’ findings in T1D [14, 25, 32]. Measures of skin fluorescence in the DCCT were no longer related to complications after adjustment to the mean HbA_1c_ [33], which suggested that the relation with complications is supported by metabolic memory, although a major part of the variance of skin fluorescence is not explained by conventional factors such as HbA_1c_, age and GFR [34]. However, a more recent work has shown that skin fluorescence could be related to subclinical cardiovascular disease independent of duration of diabetes and HbA_1c_ [18], possibly due to oxidative stress. Taken together these data indicate new therapeutic strategies for reducing diabetic complications. The clinical review of Goh and Cooper summarized the pharmalogical options that could modulate either AGE ligands accumulation or AGE-RAGE interaction [35].

Our study has some limitations. The inclusion of patients with longstanding T1D (mean duration: 21 years), often treated by medication acting on the Renin-Angiotensin System, was logical to study “hard” complications as MVE, but it may have precluded the finding of earlier events such as microalbuminuria as reported in recent and well-controlled patients with T2D [10]: the incidence of microalbuminuria is known to naturally decline after 20 years of diabetes [36]. The limited number of patients and the percentage of lost subjects represent another limitation. Moreover, the patients experienced few events over the follow-up, with thus large confidence intervals for the association between SAF and events.

## Conclusions

SAF that is strongly related to tissues biopsy levels of AGEs, has value for predicting cardiorenal outcome in our patients with T1D. This relation is independent of the usual risk factors, which supports that long-term accumulation of AGEs in tissues contributes to diabetic complications, especially nephropathy. The simple, cheap, and quick measurement of SAF makes it a good candidate for the screening of patients at risk of diabetic nephropathy.

## Abbreviations

SAF: skin autofluorescence
T1D: type 1 diabetes
MVE: macrovascular events
eGFR: estimated glomerular filtration rate
AER: albumin excretion rate
CKD: chronic kidney disease
GFR: glomerular filtration rate
AGEs: advanced glycation end-products
T2D: type 2 diabetes
AU: arbitrary unit

## References

[1] Lind M, Svensson AM, Kosiborod M, Gudbjörnsdottir S, Pivodic A, Wedel H, Dahlqvist S, Clements M, Rosengren A (2014). Glycemic control and excess mortality in type 1 diabetes. N Engl J Med.

[2] Groop PH, Thomas MC, Moran JL, Wadèn J, Thorn LM, Mäkinen VP, Rosengård-Bärlund M, Saraheimo M, Hietala K, Heikkilä O, Forsblom C, FinnDiane Study Group (2009). The presence and severity of chronic kidney disease predicts all-cause mortality in type 1 diabetes. Diabetes.

[3] Chuyen NV (2006). Toxicity of the AGEs generated from the Maillard reaction: on the relationship of food-AGEs and biological-AGEs. Mol Nutr Food Res.

[4] Huebschmann AG, Regensteiner JG, Vlassara H, Reusch JE (2006). Diabetes and advanced glycoxidation end products. Diabetes Care.

[5] Daroux M, Prevost G, Maillard-Lefebvre H, Gaxatte C, D’Agati VD, Schmidt AM, Boulanger E (2010). Advanced glycation end-products: implications for diabetic and non-diabetic nephropathies. Diabetes Metab..

[6] Nin JW, Jorsal A, Ferreira I, Schalkwijk CG, Prins MH, Parving HH, Tarnow L, Rossing P, Stehouwer CD (2011). Higher plasma levels of advanced glycation end products are associated with incident cardiovascular disease and all-cause mortality in type 1 diabetes: a 12 year follow-up study. Diabetes Care.

[7] Genuth S, Sun W, Cleary P, Sell DR, Dahms W, Malone J, Sivitz W, Monnier VM, DCCT Skin Collagen Ancillary Study Group (2005). Glycation and carboxymethyllysine levels in skin collagen predict the risk of future 10-year progression of diabetic retinopathy and nephropathy in the diabetes control and complications trial and epidemiology of diabetes interventions and complications participants with type 1 diabetes. Diabetes.

[8] Monnier VM, Sun W, Gao X, Sell DR, Cleary PA, Lachin JM, Genuth S, DCCT/EDIC Research Group (2015). Skin collagen advanced glycation endproducts (AGEs) and the long-term progression of sub-clinical cardiovascular disease in type 1 diabetes. Cardiovasc Diabetol..

[9] Meerwaldt R, Hartog JW, Graaff R, Huisman RJ, Links TP, den Hollander NC, Thorpe SR, Baynes JW, Navis G, Gans RO, Smit AJ (2005). Skin autofluorescence, a measure of cumulative metabolic stress and advanced glycation end products, predicts mortality in hemodialysis patients. J Am Soc Nephrol.

[10] Gerrits EG, Lutgers HL, Kleefstra N, Graaff R, Groenier KH, Smit AJ, Gans RO, Bilo HJ (2008). Skin autofluorescence: a tool to identify type 2 diabetic patients at risk for developing microvascular complications. Diabetes Care.

[11] Lutgers HL, Gerrits EG, Graaff R, Links TP, Sluiter WJ, Gans RO, Bilo HJ, Smit AJ (2009). Skin autofluorescence provides additional information to the UK Prospective Diabetes Study (UKPDS) risk score for the estimation of cardiovascular prognosis in type 2 diabetes mellitus. Diabetologia.

[12] Genevieve M, Vivot A, Gonzalez C, Raffaitin C, Barberger-Gateau, PGin H, Rigalleau V (2013). Skin autofluorescence is associated with past glycaemic control and complications in type 1 diabetes mellitus. Diabetes Metab..

[13] Krolewski AS, Niewczas MA, Skupien J, Gohda T, Smiles A, Eckfeldt JH, Doria A, Warram JH (2014). Early progressive renal decline precedes the onset of microalbuminuria and its progression to macroalbuminuria. Diabetes Care.

[14] Araszkiewicz A, Naskret D, Zozulinska-Ziolkiewicz D, Pilacinski S, Uruska A, Grzelka A, Wegner M, Wierusz-Wysocka B (2015). Skin autofluorescence is associated with carotid intima-media thickness, diabetic microangiopathy, and long-lasting metabolic control in type 1 diabetic patients. Results from Poznan Prospective Study. Microvasc Res.

[15] Llauradó G, Ceperuelo-Mallafré V, Vilardell C, Simó R, Gil P, Cano A, Vendrell J, González-Clemente JM (2014). Advanced glycation end products are associated with arterial stiffness in type 1 diabetes. J Endocrinol.

[16] Januszewski AS, Sachithanandan N, Karschimkus C, O’Neal DN, Yeung CK, Alkatib N, Jenkins AJ (2012). Non-invasive measures of tissue autofluorescence are increased in Type 1 diabetes complications and correlate with a non-invasive measure of vascular dysfunction. Diabet Med.

[17] Conway B, Edmundowicz D, Matter N, Maynard J, Orchard T (2010). Skin fluorescence correlates strongly with coronary artery calcification severity in type 1 diabetes. Diabetes Technol Ther..

[18] Sell DR, Sun W, Gao X, Strauch C, Lachin JM, Cleary PA, Genuth S, DCCT/EDIC Research Group (2016). Monnier VM. Skin collagen fluorophore LW-1 versus skin fluorescence as markers for the long-term progression of subclinical macrovascular disease in type 1 diabetes. Cardiovasc Diabetol..

[19] Conway BN, Aroda VR, Maynard JD, Matter N, Fernandez S, Ratner RE, Orchard TJ (2012). Skin intrinsic fluorescence is associated with coronary artery disease in individuals with long duration of type 1 diabetes. Diabetes Care.

[20] Nin JW, Ferreira I, Schalkwijk CG, Prins MH, Chaturvedi N, Fuller JH, Stehouwer CD, EURODIAB Prospective Complications Study Group (2009). Levels of soluble receptor for AGE are cross-sectionally associated with cardiovascular disease in type 1 diabetes, and this association is partially mediated by endothelial and renal dysfunction and by low-grade inflammation: the EURODIAB Prospective Complications Study. Diabetologia.

[21] Fokkens BT, Smit AJ (2016). Skin fluorescence as a clinical tool for non-invasive assessment of advanced glycation and long-term complications of diabetes. Glycoconj J.

[22] Meerwaldt R, Graaff R, Oomen PH, Links TP, Jager JJ, Alderson NL, Thorpe SR, Baynes JW, Gans RO, Smit AJ (2004). Simple non-invasive assessment of advanced glycation endproduct accumulation. Diabetologia.

[23] Hofmann B, Jacobs K, Navarrete Santos A, Wienke A, Silber RE, Simm A (2015). Relationship between cardiac tissue glycation and skin autofluorescence in patients with coronary artery disease. Diabete Metab.

[24] Tanaka K, Nakayama M, Kanno M, Kimura H, Watanabe K, Tani Y, Kusano Y, Suzuki H, Hayashi Y, Asahi K, Sato K, Miyata T, Watanabe T (2013). Skin autofluorescence is associated with the progression of chronic kidney disease: a prospective observational study. PLoS One.

[25] Sugisawa E, Miura J, Iwamoto Y, Uchigata Y (2013). Skin autofluorescence reflects integration of past long-term glycemic control in patients with type 1 diabetes. Diabetes Care.

[26] Tanaka K, Tani Y, Asai J, Nemoto F, Kusano Y, Suzuki H, Hayashi Y, Asahi K, Katoh T, Miyata T, Watanabe T (2011). Skin autofluorescence is associated with renal function and cardiovascular diseases in pre-dialysis chronic kidney disease patients. Nephrol Dial Transplant.

[27] Sternberg M, M’bemba J, Urios P, Borsos AM, Selam JL, Peyroux J, Slama G (2016). Skin collagen pentosidine and fluorescence in diabetes were predictors of retinopathy progression and creatininemia increase already 6 years after punch-biopsy. Clin Biochem.

[28] de Boer IH, Afkarian M, Rue TC, Cleary PA, Lachin JM, Molitch ME, Steffes MW, Sun W, Zinman B, Diabetes Control and Complications Trial/Epidemiology of Diabetes Interventions and Complications (DCCT/EDIC) Research Group (2014). Renal outcomes in patients with type 1 diabetes and macroalbuminuria. J Am Soc Nephrol.

[29] Beisswenger PJ, Howell SK, Russell GB, Miller ME, Rich SS, Mauer M (2013). Early progression of diabetic nephropathy correlates with methylglyoxal-derived advanced glycation end products. Diabetes Care.

[30] Rajaobelina K, Cougnard-Gregoire A, Delcourt C, Gin H, Barberger-Gateau P, Rigalleau V (2015). Autofluorescence of skin advanced glycation end products: marker of metabolic memory in elderly population. J Gerontol A Biol Sci Med Sci.

[31] Lind M, Odén A, Fahlén M, Eliasson B (2010). The shape of the metabolic memory of HbA1c: re-analysing the DCCT with respect to time-dependent effects. Diabetologia.

[32] Banser A, Naafs JC, Hoorweg-Nijman JJ, van de Garde EM, van der Vorst MM (2016). Advanced glycation end products, measured in skin, vs. HbA1c in children with type 1 diabetes mellitus. Pediatr Diabetes..

[33] Orchard TJ, Lyons TJ, Cleary PA, Braffett BH, Maynard J, Cowie C, Gubitosi-Klug RA, Way J, Anderson K, Barnie A, Villavicencio S, DCCT/EDIC Research Group (2013). The association of skin intrinsic fluorescence with type 1 diabetes complications in the DCCT/EDIC study. Diabetes Care.

[34] Cleary PA, Braffett BH, Orchard T, Lyons TJ, Maynard J, Cowie C, Gubitosi-Klug RA, Way J, Anderson K, Barnie A, Villavicencio S, DCCT/EDIC Research Group (2013). Clinical and technical factors associated with skin intrinsic fluorescence in subjects with type 1 diabetes from the Diabetes Control and Complications Trial/Epidemiology of Diabetes Interventions and Complications Study. Diabetes Technol Ther..

[35] Goh SY, Cooper ME (2008). Clinical review: The role of advanced glycation end products in progression and complications of diabetes. J Clin Endocrinol Metab.

[36] Borch-Johnsen K (1993). Epidemiology of microangiopathy in type 1 diabetes mellitus. A review. Diabete Metab.

